# New ternary inverter with memory function using silicon feedback field-effect transistors

**DOI:** 10.1038/s41598-022-17035-z

**Published:** 2022-07-28

**Authors:** Jaemin Son, Kyoungah Cho, Sangsig Kim

**Affiliations:** Department of Electrical Engineering, Korea University, 145 Anam-ro, Seongbuk-gu, Seoul, 02841 Republic of Korea

**Keywords:** Electrical and electronic engineering, Electronics, photonics and device physics

## Abstract

In this study, we present a fully complementary metal–oxide–semiconductor-compatible ternary inverter with a memory function using silicon feedback field-effect transistors (FBFETs). FBFETs operate with a positive feedback loop by carrier accumulation in their channels, which allows to achieve excellent memory characteristics with extremely low subthreshold swings. This hybrid operation of the switching and memory functions enables FBFETs to implement memory operation in a conventional CMOS logic scheme. The inverter comprising p- and n-channel FBFETs in series can be in ternary logic states and retain these states during the hold operation owing to the switching and memory functions of FBFETs. It exhibits a high voltage gain of approximately 73 V/V, logic holding time of 150 s, and reliable endurance of approximately 10^5^. This ternary inverter with memory function demonstrates possibilities for a new computing paradigm in multivalued logic applications.

## Introduction

Over the past five decades, electronic devices have been continuously scaled down to a few nanometers to satisfy the rapidly increasing demand for high performance and density^1,2^. However, the performance of current electronic systems has been limited by several drawbacks, including long signal delays and high-power consumption owing to wire resistances and capacitances^3–5^. To overcome these drawbacks, multivalued logic (MVL) systems utilizing more than two logic states have garnered considerable attention^6–8^. In MVL systems, the density of information can substantially increase, compared to conventional binary logic systems, because discrete devices in MVL systems hold more than two datasets. Accordingly, fewer devices and wires are required in MVL systems to process the same amount of data than those of conventional binary logic systems. To date, significant efforts have been made to realize MVL systems using various devices, such as ternary complementary metal–oxide–semiconductor (T-CMOS)^9–11^, carbon nanotube field-effect transistors (CNTFETs)^12–14^, organic semiconductors^15,16^, and 2D materials^17–19^. In particular, T-CMOS, which uses a junction band-to-band tunneling current to create a third state, has shown the advantages of CMOS process compatibility and power scalability. However, these MVL systems have chronic problems of leakage current or increase in the number of devices to use the multivalued state. The way to overcome these issues is to adopt the logic-in-memory function in the MVL systems by using switchable-memory devices. In a logic-in-memory computing system, the memory and logic operations are merged in a single basic device structure^20,21^. Accordingly, the logic-in-memory computing system can substantially reduce the power consumption and circuit density.

Recently, feedback field-effect transistors (FBFETs) have opened up the possibilities for logic-in-memory computing systems owing to their switchable-memory characteristics^22–27^. FBFETs are one of the novel FETs that allow the applications in both ternary logic and logic-in-memory computing systems. FBFETs operate with a positive feedback loop by carrier accumulation in their channels, which allows to achieve excellent memory characteristics with extremely low subthreshold swings (*SS*s)^28,29^. This hybrid operation of the switching and memory functions enables FBFETs to implement memory operation in a conventional CMOS logic scheme^30^. Therefore, in this study, we introduce a fully CMOS-compatible ternary inverter with a memory function that consists of a p-channel FBFET (p-FBFET) and an n-channel FBFET (n-FBFET) in series. The ternary inverter exhibits three distinguished logic states, and it can retain these states owing to the memory operation of the FBFETs.

## Experimental section

### Device fabrication

FBFETs were fabricated on a silicon-on-insulator wafer using a fully CMOS conductor compatible top-down method. A silicon active layer at a depth of 340 nm was formed through stepper photolithography and an anisotropic dry etching process. N-type (for p-FBFETs) and p-type wells (for n-FBFETs) were formed by implantation of P^+^ 3 × 10^13^ cm^−2^ at 60 keV and BF_2_^+^ 5 × 10^12^ cm^−2^ at 40 keV, respectively. A well drive-in was performed at 1100 °C for 30 min. A silicon dioxide (SiO_2_) gate dielectric with a thickness of 22 nm was thermally grown at 850 °C, and a polysilicon gate was formed on top of a channel using a low-temperature chemical vapor deposition (LPCVD) and photolithography. Tetraethyl orthosilicate gate sidewall spacers with a length of approximately 200 nm were formed using LPCVD. BF_2_^+^ ions at a dose of 6 × 10^13^ cm^−2^ at 40 keV and P^+^ ions with a dose of 1 × 10^14^ cm^−2^ at 60 keV were implanted to form p-type nongated (for p-FBFET) and n-type nongated region (for n-FBFET) regions, respectively. In addition, the p^+^ drain contact regions were heavily doped with BF_2_^+^ ions at a dose of 3 × 10^15^ cm^−2^ at 30 keV. The n^+^ source contact regions were heavily doped with P^+^ ions at a dose of 3 × 10^15^ cm^−2^ at 100 keV for the p-FBFET and at a dose of 4 × 10^15^ cm^−2^ at 50 keV for n-FBFET. Subsequently, the wafer was annealed at 1000 °C for 30 min and then at 1050 °C for 30 s using a rapid thermal annealing system to activate the implanted dopants. Finally, the drain, source, and gate electrodes were made of Ti/TiN/Al/TiN metal alloy using sputtering and photolithography.

### Measurements

The electrical properties were measured at room temperature using an Agilent HP4155C semiconductor parameter analyzer, a Tektronix AFG 31000 arbitrary function generator, and a Tektronix MDO3054 mixed-domain oscilloscope. The cross-section image of the FBFET was obtained using transmission electron microscopy (TEM; Tecnai G2 F20, FEI).

## Results and discussion

**Figure 1:**
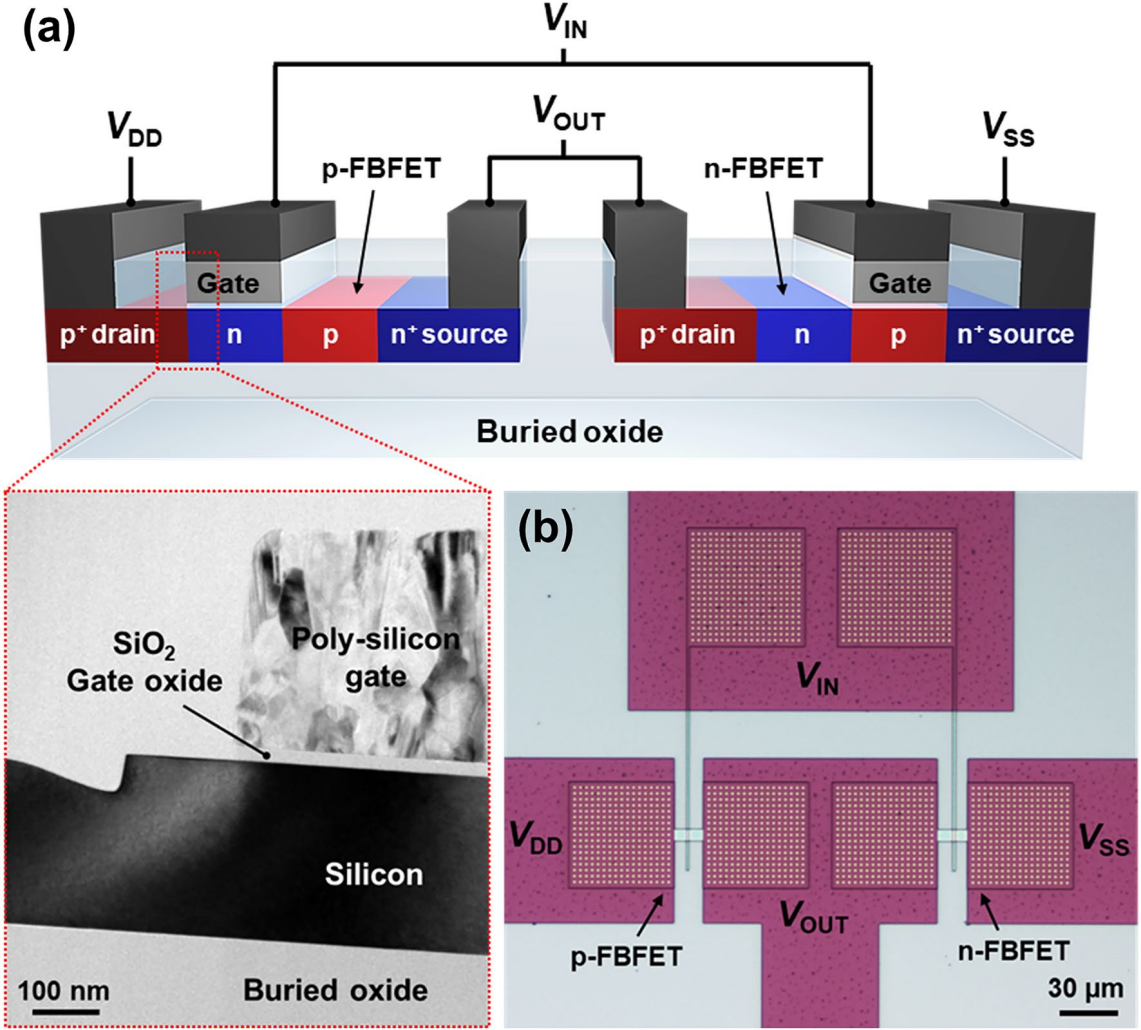
(**a**) Schematic of three-dimensional view, and (**b**) optical top-view image of the ternary inverter comprising the p- and n-FBFET. The bottom left inset shows cross-sectional TEM image of a FBFET.

A three-dimensional schematic and optical image of a ternary inverter comprising single-gated n- and p-FBFETs connected in series are shown in Fig. 1a,b, respectively. The basic structure of FBFETs consists of a heavily doped p^+^ drain region, heavily doped n^+^ source region, and p-n channel region. Although the p-n-p-n energy band structure of the FBFET is similar to those of tunneling devices, the band-to-band tunneling (BTBT) is not a significant factor in the FBFET operation. In the FBFET, the forward bias is used to generate the positive feedback mechanism, whereas tunneling devices use reverse bias to operate with BTBT mechanism. As a result, our device does not suffer from degradation in switching speed caused by the BTBT. The channel lengths of the n- and p-FBFET were 5 and 4 µm, respectively, and different channel lengths were used to achieve identical channel resistances for these FBFETs in the on state. The source of the n-FBFET is connected to *V*_SS_, and the drain of the p-FBFET is connected to *V*_DD_ as the power supply voltage. The gates of the n- and p-FBFET were shared as an input node, and the drain of the n-FBFET and source of the p-FBFET were shared as an output node.

**Figure 2:**
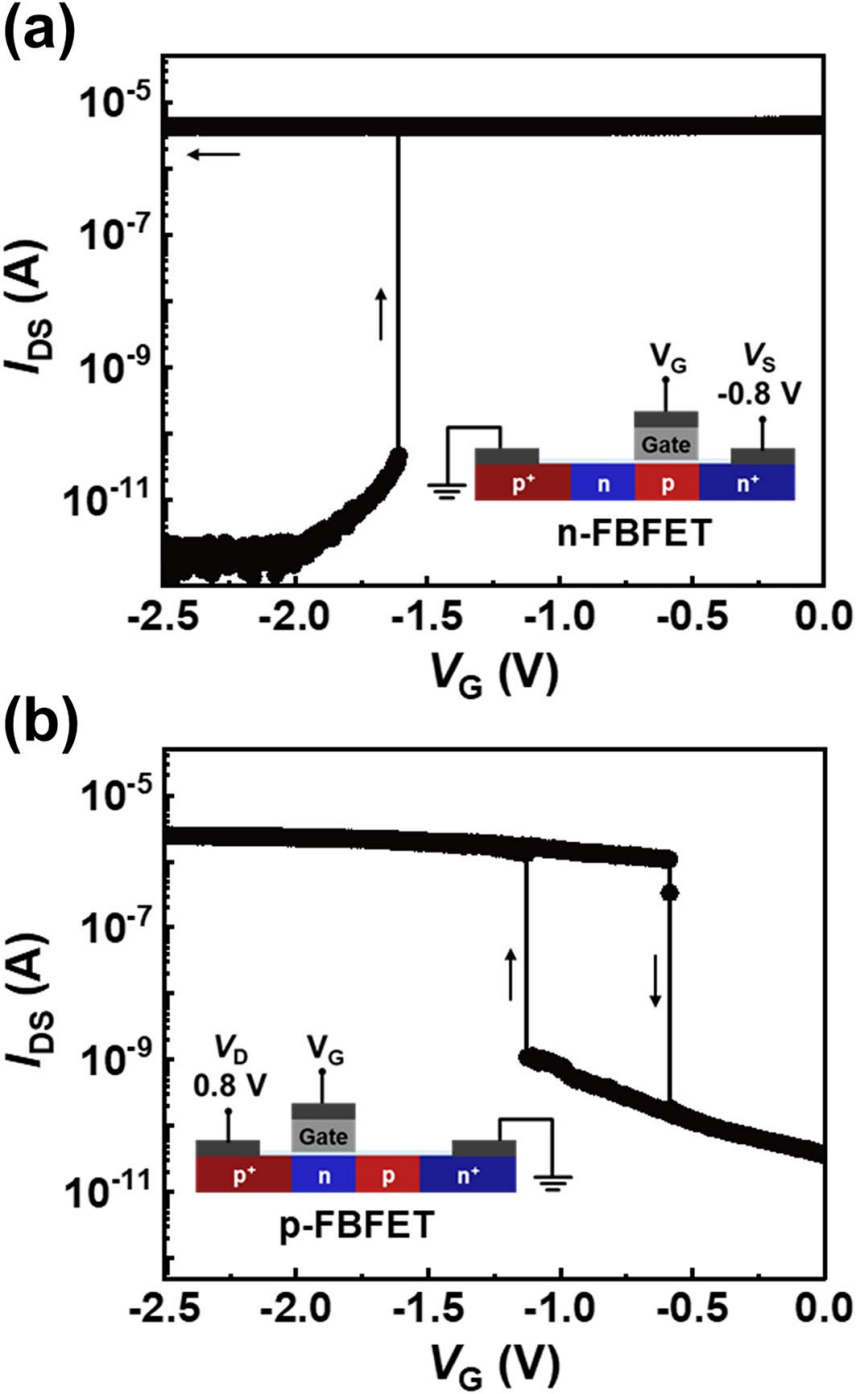
Representative transfer characteristics of (**a**) n- and (**b**) p-FBFET.

Figure 2 shows the representative transfer characteristics of an n-FBFET at a source voltage (*V*_S_) of − 0.8 V and at a drain voltage (*V*_D_) of 0.0 V, and a p-FBFET at a *V*_D_ of 0.8 V and a *V*_S_ of 0.0 V. During the double gate voltage (*V*_G_) sweep, both FBFETs exhibit steep switching and hysteresis characteristics owing to the positive feedback loop in their channel regions. The hysteresis characteristics allow the bistable states, demonstrating that these FBFETs operate as memory devices. Hence, the positive-feedback mechanism enables both the switching and memory functions in FBFETs. The values of *SS*s obtained using *SS* = d*V*_GS_/dlog(|*I*_DS_|) are extremely low; approximately 0.60 and 0.97 mV/dec for the n- and p-FBFET, respectively. The memory window (MW), which is determined using the difference between latch-up and -down voltages, is 0.59 V for the p-FBFET at *V*_D_ = 0.8 V and *V*_S_ = 0.0 V. Moreover, the n-FBFET has an open MW at *V*_S_ = − 0.8 V and *V*_D_ = 0.0 V.

**Figure 3:**
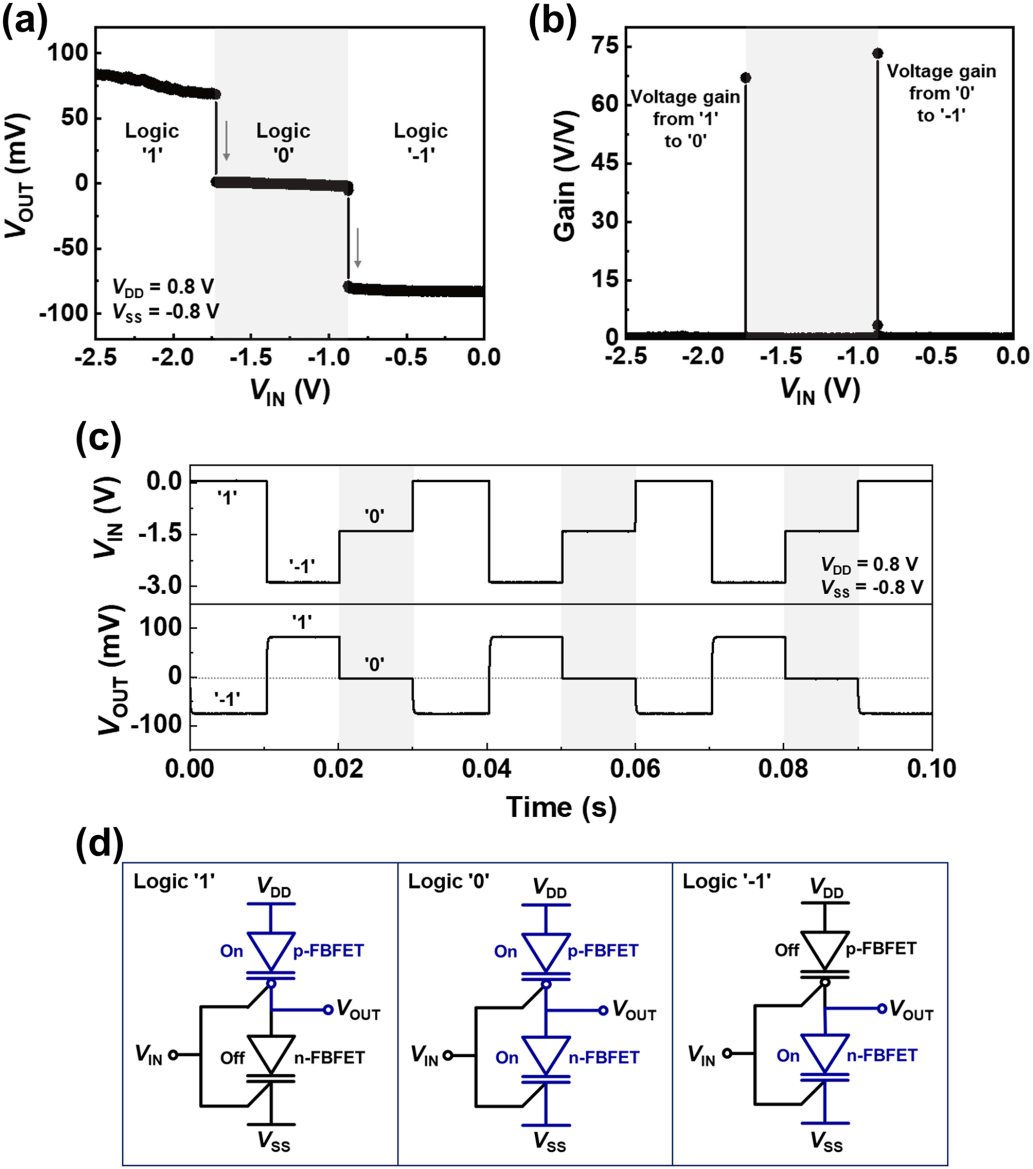
(**a**) Voltage transfer characteristics of a ternary inverter, and (**b**) corresponding voltage gains. (**c**) Logic operation (‘1’, ‘0’, and ‘− 1’) under dynamic condition. (**d**) Schematic for logic ‘1’, ‘0’, and ‘− 1’ operation.

Next, we investigated the ternary logic operation of the introduced inverter. Figure 3a shows the voltage transfer characteristics as a function of the input voltage at *V*_DD_ = 0.8 V and *V*_SS_ = − 0.8 V. When the input voltage (*V*_IN_) is swept from − 2.5 to 0.0 V with a voltage step of 1 mV, *V*_OUT_ exhibits three distinguishable logic states; high level (logic ‘1’), intermediate level (logic ‘0’), and low level (logic ‘− 1’). A stable *V*_OUT_ value in each of the three logic states was determined by the ratio of the channel resistances of the n- and p-FBFET. Moreover, an extremely low *SS* of FBFETs allows sharp voltage transitions with input voltages. The voltage gain in the transition from the logic ‘1’ (‘0’) to the logic ‘0’ (‘− 1’) is ~ 67 V/V (~ 73 V/V), as shown in Fig. 3b. The dynamic *V*_OUT_ characteristics at 100 Hz are shown in Fig. 3c. The input frequency of 100 Hz is the maximum frequency to show clearly the output response; the output response is delayed along with the increase in the input frequency (see supplementary information). *V*_OUT_ transitions are clearly visible with stepwise three-level *V*_IN_ pulses (0.0 → − 1.5 → − 3.0 V). The abnormal *V*_IN_ range shown in Fig. 3 is deeply concerned with modulating the potential barrier height in the channel region; the potential barrier height is adjusted by the gate workfunction, channel doping concentration, and etc. Hence, workfunction matching and lightly doping on the channel may contribute to the positively shift of the abnormal *V*_IN_ range.

To further investigate the logic operating mechanism of the proposed ternary inverter, schematic diagrams operating in logic ‘1’, ‘0’, and ‘− 1’ states are depicted in Fig. 3d. In the logic ‘1’ state with *V*_IN_ ≤ − 1.7 V, p-FBFET is in the on state and provides a low resistive path between *V*_DD_ and *V*_OUT_. However, in the logic ‘− 1’ state with *V*_IN_ ≥ − 0.8 V, n-FBFET is in the on state and provides a low resistive path between *V*_SS_ and *V*_OUT_. Therefore, the output node is set to a positive voltage in the logic ‘1’ state and a negative voltage in the logic ‘− 1’ state. The additional intermediate level corresponds to logic ‘0’ state, which stems from the region where the n- and p-FBFET are simultaneously in the on states (− 1.7 V ≤ *V*_IN_ ≤ − 0.8 V). The ratio of the channel resistances of the n- and p-FBFET in the on states approaches 1, and thereby a stable intermediate value of approximately 0 V appears in the output node. On the other hand, the decrease in the output voltage can be explained in terms of charging in the FBFET and the output impedance limitation of our oscilloscope. Due to charging in the FBFET, the FBFET acts as a capacitor and causes the output voltage drop. The maximum output impedance of our oscilloscope is 1 MΩ that is close to the on-state channel resistance of the FBFETs. Accordingly, the output voltage was divided between the FBFET channel resistance and the oscilloscope impedance.

**Figure 4:**
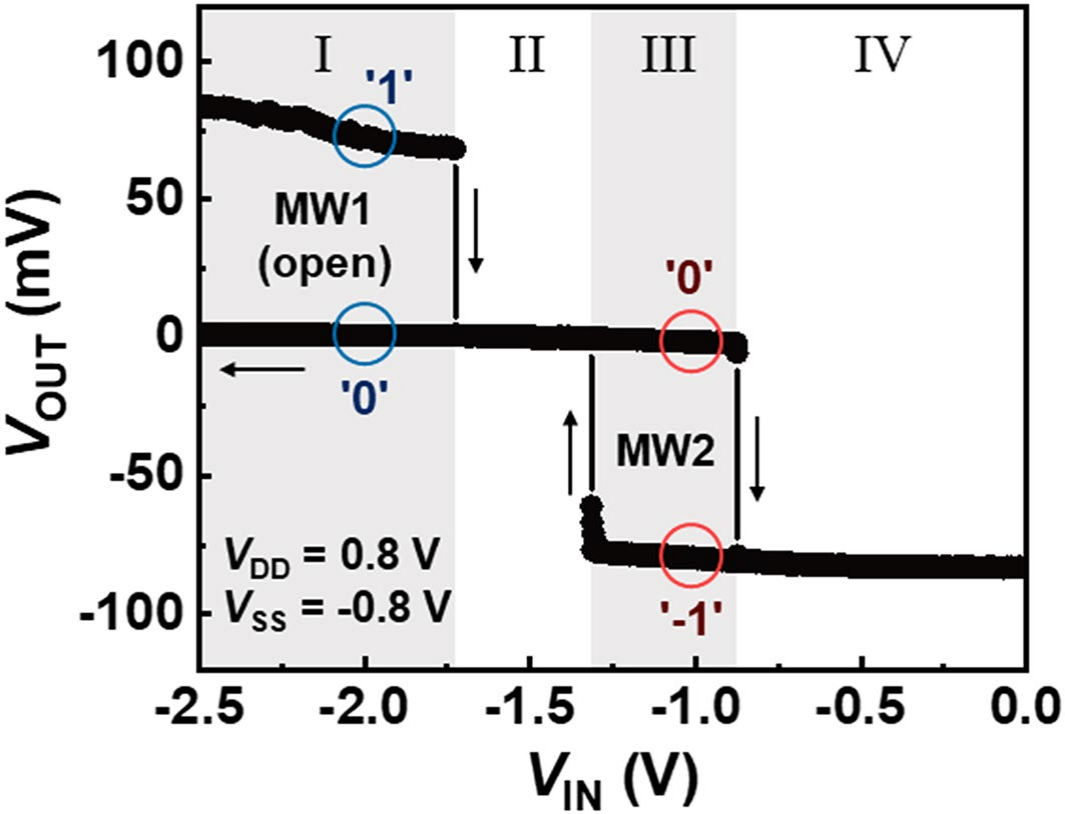
Voltage hysteresis characteristics of the proposed ternary inverter, indicating two different MWs (MW1 and MW2).

Figure 4 shows the voltage hysteresis characteristics (VHC) of the ternary inverter. The hysteresis curves are divided into four regions: I, II, III, and IV. In these regions, the on/off states of the n- and p-FBFET and the logic ‘0’/‘− 1’ states of the ternary inverter are examined using the representative transfer characteristics shown in Fig. 2. In region I (*V*_IN_ ≤ − 1.7 V), the n-FBFET has bistable states depending on the *V*_IN_ sweep directions, and the p-FBFET is in the on state. Therefore, the open MW (MW1) in the VHC, which shows the logic ‘1’ and ‘0’ states, results from the MW of the n-FBFET. In region II (− 1.7 V ≤ *V*_IN_ ≤ − 1.3 V), both the n- and p-FBFET are in the on state. In region III (− 1.3 V ≤ *V*_IN_ ≤ − 0.8 V), the secondary MW (MW2) appears in the VHC, which agrees with the MW of the p-FBFET. Meanwhile, the n-FBFET is in the on state, and thus MW2 shows the logic ‘0’ and ‘− 1’ states. In region IV (*V*_IN_ ≥ − 0.8 V), the n- and p-FBFET are in the on and off states, respectively. The two MWs in regions I and III are the unique characteristics of the proposed ternary inverter that is owing to the inherent hysteresis curve (*I*_DS_–*V*_GS_) of FBFETs.

**Figure 5:**
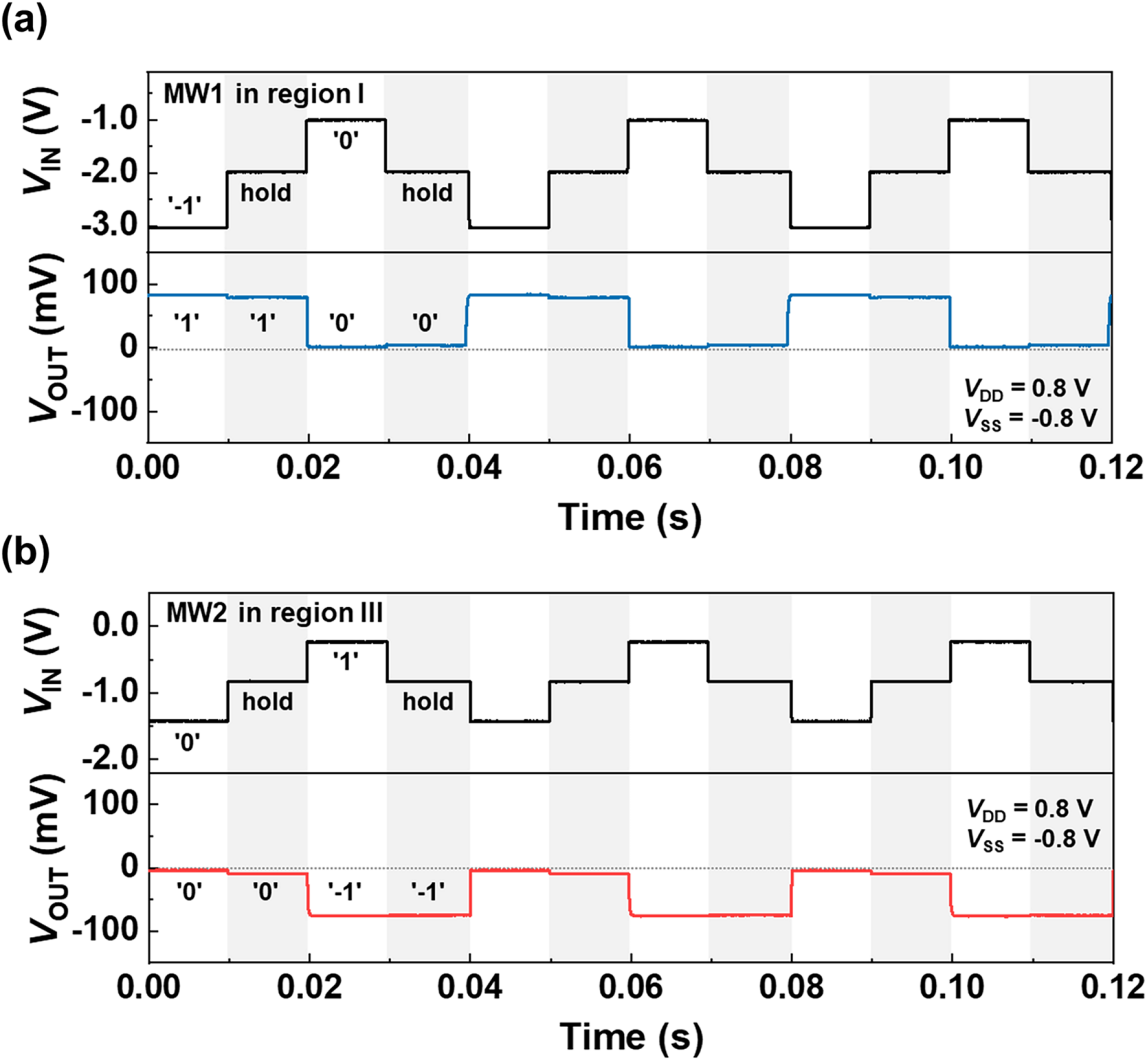
Holding operation of (**a**) logic ‘1’ and ‘0’ states in MW1, and (**b**) logic ‘0’ and ‘− 1’ states in MW2 under dynamic condition.

Figure 5a,b show the memory operation of the ternary inverter under the dynamic condition at *V*_DD_ = 0.8 V and *V*_SS_ = − 0.8 V. A sequence of the write and hold operations of logic ‘1’ and ‘0’ states using MW1 (region I) are depicted in Fig. 5a. The write conditions of input voltages for logic ‘1’ and ‘0’ are set to − 3.0 and − 1.0 V, respectively. The writing operation of logic ‘1’ is performed successfully by the pulsed *V*_IN_, even though MW1 is opened. Under dynamic conditions, a relatively high negative *V*_IN_ (− 3.0 V) pulse extricates the accumulated holes in the gated channel region of the n-FBFET^22^. Accordingly, the positive feedback loop is eliminated, and the n-FBFET is in the off state owing to the emission of accumulated holes. Thus, the output of the ternary inverter reveres from the logic ‘0’ state to the logic ‘1’ state. After the write operation, the holding process was performed by sensing the difference in the *V*_OUT_ of logic ‘1’ and ‘0’ states at *V*_IN_ = − 2.0 V, which is within the range of MW1. During the hold operation, the ternary inverter stably maintains in the logic ‘1’ and ‘0’ states. Moreover, the memory operation of logic states ‘0’ and ‘− 1’ using MW2 (region III) is depicted in Fig. 5b. When a *V*_IN_ of − 1.5 V (− 0.3 V) is applied, the ternary inverter reverses the logic states from ‘− 1’ (‘0’) to ‘0’ (‘− 1’). For the holding operation of logic ‘0’ and ‘− 1’ states, *V*_IN_ is set to − 0.9 V, which is within the range of MW2. During the hold operation, FBFETs maintained their on or off states; thus, the ternary inverter memorizes the logic ‘0’ or ‘− 1’ states. In our ternary inverter, the memory characteristics allow to maintain the ternary output voltage without any additional circuit. Accordingly, the ternary inverter can be embedded in the CPU and it can replace the volatile memory block or D-latch circuit.

**Figure 6:**
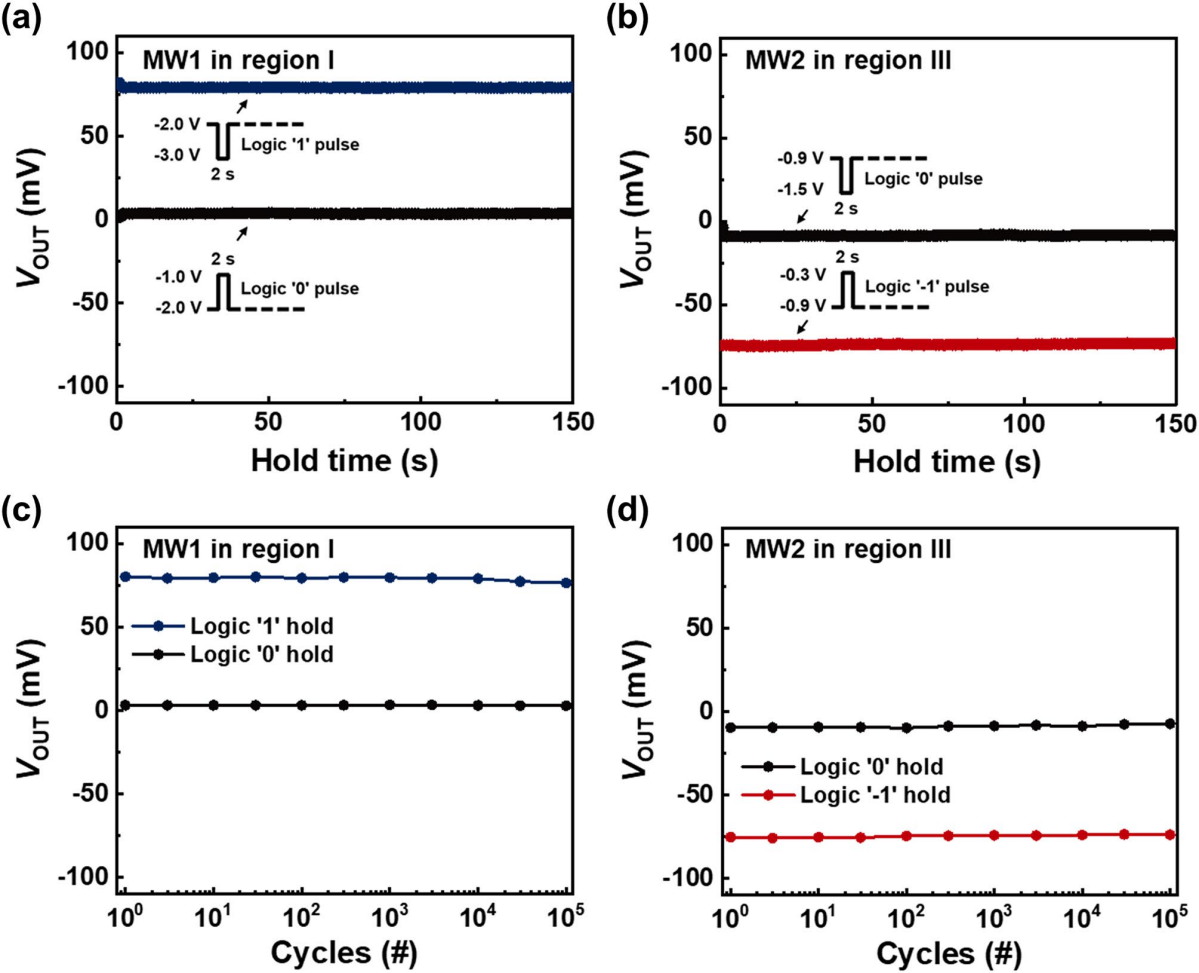
(**a**,**b**) Logic retention, and (**c**,**d**) endurance characteristics of the ternary inverter in each of MW1 and MW2.

The retention properties of the proposed ternary inverter using each of the separate MWs (MW1 and MW2) are shown in Fig. 6a,b. After write pulses with a time width of 2 s are applied, the ternary inverter stably maintains the logic ‘1’ or ‘0’ states at *V*_IN_ = − 2.0 V and the logic ‘0’ or ‘− 1’ states at *V*_IN_ = − 0.9 V for a holding time of 150 s. Figure 6c,d show the endurance characteristics of the ternary inverter as a function of the number of write/hold memory cycles. During the endurance evaluation, the pulse cycles of the memory operations using MW1 (Fig. 6c) and MW2 (Fig. 6d) are the same as those in Fig. 5a,b, respectively. The presented ternary inverter showed reliable characteristics even after 10^5^ cycles of memory operations, implying that the degradation of the three logic states of the ternary inverter is negligible during memory operations.

## Conclusion

In this study, we introduced a fully CMOS-compatible ternary inverter that operates with a memory function using FBFETs. The ternary inverter exhibited three logic states of ‘− 1’, ‘0’, and ‘1’ with a high voltage gain of approximately 73 V/V owing to the positive feedback mechanism. Moreover, the ternary inverter retained the logic states during the holding operation, and exhibited a logic holding time and reliable endurance of approximately 150 s and 10^5^, respectively. Hence, the proposed ternary inverter provides possibilities for a new computing paradigm in multivalued logic applications using its memory function.

## Supplementary Information

Supplementary Figure S1.

## Data Availability

All data generated during this study are included in this published article.
