# Supplementary material for: New ternary inverter with memory function using silicon feedback field-effect transistors
Source: Sci Rep. 2022 Jul 28;12:12907. doi: 10.1038/s41598-022-17035-z (PMC9334607; doi:10.1038/s41598-022-17035-z)
Supplement: Supplementary file 1 — Supplementary Figure S1. [file 41598_2022_17035_MOESM1_ESM.docx]

Supplementary Information

New ternary inverter with memory function using silicon feedback field-effect transistors

*Jaemin Son, Kyoungah Cho, and Sangsig Kim*^*^

**AFFILIATIONS**

Department of Electrical Engineering, Korea University, 145 Anam-ro, Seongbuk-gu,
Seoul 02841, Republic of Korea

^*^Corresponding author. Tel: +82-2-3290-3245; Fax: +82-2-3290-3894

E-mail address: sangsig@korea.ac.kr

**Output characteristics of the ternary inverter according to the input frequency**

Figure S1 shows the output characteristics of the ternary inverter for different input frequencies (10 Hz to 10 kHz), indicating that the output response is delayed along with the increase of the input frequency.


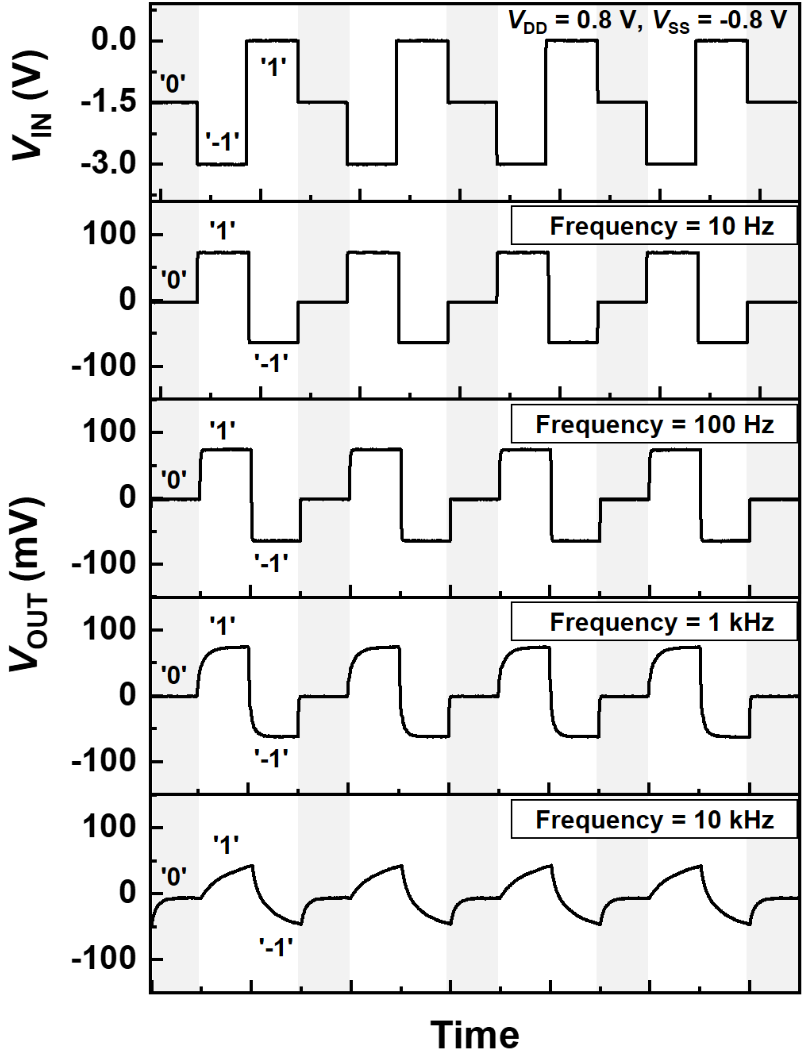


**Figure S1** Output characteristics of the ternary inverter according to the input frequency.
